# Uridine Adenosine Tetraphosphate-Induced Coronary Relaxation Is Blunted in Swine With Pressure Overload: A Role for Vasoconstrictor Prostanoids

**DOI:** 10.3389/fphar.2018.00255

**Published:** 2018-03-26

**Authors:** Zhichao Zhou, Inge M. Lankhuizen, Heleen M. van Beusekom, Caroline Cheng, Dirk J. Duncker, Daphne Merkus

**Affiliations:** ^1^Division of Experimental Cardiology, Department of Cardiology, Thoraxcenter, Erasmus Medical Center, University Medical Center Rotterdam, Rotterdam, Netherlands; ^2^Cardiovascular Research School Erasmus University Rotterdam, Erasmus Medical Center, University Medical Center Rotterdam, Rotterdam, Netherlands; ^3^Division of Cardiology, Department of Medicine, Karolinska University Hospital, Karolinska Institutet, Solna, Sweden; ^4^Department of Nephrology and Hypertension, University Medical Center Utrecht, Utrecht, Netherlands

**Keywords:** Up_4_A, coronary, microcirculation, purinergic receptor, hypertension, prostanoid

## Abstract

Plasma levels of the vasoactive substance uridine adenosine tetraphosphate (Up_4_A) are elevated in hypertensive patients and Up_4_A-induced vascular contraction is exacerbated in various arteries isolated from hypertensive animals, suggesting a potential role of Up_4_A in development of hypertension. We previously demonstrated that Up_4_A produced potent and partially endothelium-dependent relaxation in the porcine coronary microvasculature. Since pressure-overload is accompanied by structural abnormalities in the coronary microvasculature as well as by endothelial dysfunction, we hypothesized that pressure-overload blunts the coronary vasodilator response to Up_4_A, and that the involvement of purinergic receptors and endothelium-derived factors is altered. The effects of Up_4_A were investigated using wire-myography in isolated coronary small arteries from Sham-operated swine and swine with prolonged (8 weeks) pressure overload of the left ventricle induced by aortic banding (AoB). Expression of purinergic receptors and endothelium-derived factors was assessed in isolated coronary small arteries using real-time PCR. Up_4_A (10^-9^ to 10^-5^ M) failed to produce contraction in isolated coronary small arteries from either Sham or AoB swine, but produced relaxation in preconstricted arteries, which was significantly blunted in AoB compared to Sham. Blockade of purinergic P1, and P2 receptors attenuated Up_4_A-induced coronary relaxation more, while the effect of P2X_1_-blockade was similar and the effects of A_2A_- and P2Y_1_-blockade were reduced in AoB as compared to Sham. mRNA expression of neither A1, A2, A3, nor P2X_1_, P2X_7_, P2Y_1_, P2Y_2_, nor P2Y_6_-receptors was altered in AoB as compared to Sham, while P2Y_12_ expression was higher in AoB. eNOS inhibition attenuated Up_4_A-induced coronary relaxation in both Sham and AoB. Additional blockade of cyclooxygenase enhanced Up_4_A-induced coronary relaxation in AoB but not Sham swine, suggesting the involvement of vasoconstrictor prostanoids. In endothelium-denuded coronary small arteries from normal swine, thromboxane synthase (TxS) inhibition enhanced relaxation to Up_4_A compared to endothelium-intact arteries, to a similar extent as P2Y_12_ inhibition, while the combination inhibition of P2Y_12_ and TxS had no additional effect. In conclusion, Up_4_A-induced coronary relaxation is blunted in swine with AoB, which appears to be due to the production of a vasoconstrictor prostanoid, likely thromboxane A_2_.

## Introduction

Hypertension and aortic stenosis result in chronic pressure-overload of the left ventricle, producing left ventricular hypertrophy, and are considered risk factors for the development of heart failure (Khatibzadeh et al., 2013). Prolonged pressure-overload has also been shown to cause structural and functional adaptations in the coronary vasculature. Coronary flow reserve is decreased and minimal coronary resistance is increased (Duncker et al., 1993; Hamasaki et al., 2000; Galderisi et al., 2001), due to increased extravascular compression of the arterioles, decreased capillary density and vascular remodeling (Breisch et al., 1986; Tomanek et al., 1986; Bache, 1988; Hamasaki et al., 2000; van den Heuvel et al., 2001; Urbieta-Caceres et al., 2011). Functional changes in the coronary vasculature mainly result from an increase in oxidative stress and endothelial dysfunction (Rodriguez-Porcel et al., 2003; Lavi et al., 2008; Alexánderson et al., 2012) resulting in an imbalance between endothelium-derived vasodilators such as Nitric Oxide (NO) and prostacyclin and endothelium-derived vasoconstrictors like endothelin and reactive oxygen species (Vanhoutte, 1996).

Uridine adenosine tetraphosphate (Up4A) was initially identified as an endothelium-derived vasoconstrictor. A role for Up_4_A in the pathogenesis of hypertension has been suggested by the observation that Up_4_A-induced vascular contraction in the renal, femoral and basilar artery is potentiated in hypertension (Matsumoto et al., 2011b). In addition, the Up_4_A plasma concentration in hypertensive subjects is significantly higher as compared to healthy subjects and positively correlates with blood pressure (Jankowski et al., 2007). Up_4_A contains both purine and pyrimidine moieties and, like other extracellular nucleotides, exerts its vasoactive influence by binding to purinergic receptors (Matsumoto et al., 2011a; Zhou et al., 2013b), a class of receptors comprising P1 (adenosine receptors) and P2 receptors, that can be further subdivided into P2X and P2Y subtypes (Ralevic and Burnstock, 1998). Subsequent studies found that the vasoactive effect of Up_4_A is not only dependent on the vascular bed but also on the species studied. Thus, Up_4_A produces potent relaxation in the healthy porcine coronary vasculature (Zhou et al., 2013b) whereas vasoconstriction is observed in response to Up_4_A in the murine coronary microcirculation (Teng et al., 2017). Interestingly, Up_4_A-induced relaxation is attenuated in the remote coronary vasculature after myocardial infarction via downregulation of P1 receptors (Zhou et al., 2013a). Although Up_4_A-induced coronary relaxation is maintained in swine with metabolic derangement, the purinergic signaling and endothelium-derived factors involved in Up_4_A responses are markedly altered (Zhou et al., 2017). Altogether, these findings indicate that the effects of Up_4_A in the porcine coronary vasculature change in cardiovascular disease.

Consequently, the first aim of the present study was to investigate whether prolonged pressure overload induced by AoB alters the response to Up_4_A in isolated porcine coronary small arteries, and to determine the purinergic receptors and endothelium-derived factors mediating this altered vascular response to Up_4_A. Interestingly, we found evidence for release of a vasoconstrictor prostanoid in response to Up_4_A in AoB. As the vasoconstrictor prostanoid thromboxane A_2_ (TxA_2_) was recently shown to be released in response to Up_4_A in renal arteries and aortas (Matsumoto et al., 2014; Zhou et al., 2015b), we further investigated whether Up_4_A can induce production of TxA_2_ in the porcine coronary microvasculature.

## Materials and Methods

### Animals

Studies were performed in accordance with the “Guiding Principles in the Care and Use of Laboratory Animals” as approved by the Council of the American Physiological Society, and with approval of the Animal Care Committee at Erasmus Medical Center Rotterdam. Fifteen Crossbred Yorkshire X Landrace swine (2 to 3-month-old, 18.5 ± 0.3 kg at the time of surgery) of either sex entered the study. Swine were housed in the animal facility in cages with a 12/12 h light/dark cycle, *ad libitum* access to water and were fed twice per day. After 1 week of daily adaptation to laboratory conditions, animals underwent either AoB (*n* = 7) or a sham procedure (Sham; *n* = 8).

### Surgery

After overnight fasting, swine were sedated with an intramuscular injection of Zoletil (Tiletamine/Zolazepam; 5 mg kg^-1^), Xylazine (2.25 mg kg^-1^), and Atropine (1 mg), and a small catheter was placed in an earvein for subsequent administration of fluid. Swine were intubated and ventilated with a mixture of oxygen and nitrogen (1:2 vol/vol), to which 2% (vol/vol) isoflurane was added to maintain anesthesia (Haitsma et al., 2001; Kappers et al., 2012). Under sterile conditions, the chest was opened via the fourth left intercostal space and fluid-filled polyvinylchloride catheters were inserted into the left ventricle (LV), only in AoB swine, and in the aortic arch (Ao) of both Sham and AoB swine, for the measurement of the pressure and blood sampling for the determination of PO_2_, PCO_2_, pH, O_2_ saturation, and hemoglobin concentration. The ascending aorta was exposed in all swine, a sterile plastic band was placed around the ascending aorta in AoB animals and gradually tightened until the systolic pressure gradient between distal aorta and proximal LV catheters reached approximately 80 mmHg. Then, the chest was closed and animals were allowed to recover, receiving analgesia (0.3 mg buprenorphine i.m.) for 2 days and antibiotic prophylaxis (25 mg kg^-1^ amoxicillin and 5 mg kg^-1^ gentamycin i.v.) for 5 days. Pressure in the proximal and distal catheters were recorded at the time of surgery, as well as 1 and 3 weeks after surgery, and the systolic pressure gradient was calculated. Due to malfunctioning of the catheters in a number of animals, pressures could not be obtained 8 weeks after the initial surgery.

Eight weeks after initial Sham and AoB surgery, animals were re-anesthetized, intubated and ventilated as described above. Pentobarbital (20 mg kg^-1^ h^-1^) was infused to induce and maintain anesthesia. A catheter was introduced via the femoral artery into the descending aorta for measurement of mean arterial pressure. A Swan Ganz catheter was inserted via the jugular vein and advanced into the pulmonary artery for measurement of PAP and CO (via thermodilution) (van Kats et al., 2000). Following thoracotomy, hearts were arrested and immediately excised and placed in cold, oxygenated Krebs bicarbonate buffer solution.

### Myograph Studies

Coronary small arteries (diameter: ∼150 μm) were dissected out from the apex of eight Sham-operated and seven AoB swine and stored overnight in cold, oxygenated Krebs bicarbonate solution of the following composition (mM): NaCl 118, KCl 4.7, CaCl_2_ 2.5, MgSO_4_ 1.2, KH_2_PO_4_ 1.2, NaHCO_3_ 25, and glucose 8.3; pH 7.4. The next day, coronary small arteries were cut into segments of ∼2 mm length and mounted in microvascular myographs (Danish Myo Technology) with separated organ baths containing 6 ml Krebs bicarbonate solution aerated with 95%O_2_/5%CO_2_ and maintained at 37°C (Batenburg et al., 2004; Zhou et al., 2013b, 2017). Changes in contractile force were recorded with a Harvard isometric transducer. Following a 30 min stabilization period, the internal diameter was set to a tension equivalent to 0.9 times the estimated diameter at 100 mmHg effective transmural pressure (Batenburg et al., 2004; Zhou et al., 2013b, 2017). At the end of the stabilization period, the vessels were exposed to 30 mM KCl twice to check the contractility. Endothelial integrity was verified by observing dilation to 10 nM substance P after preconstriction with 100 nM of the stable TxA_2_ analog U46619. Then vessels were subjected to 100 mM KCl to determine maximal vascular contraction. Thereafter, vessels were allowed to equilibrate in fresh organ bath fluid for 30 min before initiating different experimental protocols (Batenburg et al., 2004; Zhou et al., 2013b). In experiments where the effect of an antagonist on the response to Up_4_A was assessed, antagonists were added to the organ baths 30 min before preconstriction with U46619 and were present throughout the experiments. Only one protocol was executed per vessel and, within one protocol, all vessels were obtained from different animals.

### Experimental Protocols

Coronary small arteries from both Sham and AoB swine were subjected to Up_4_A in incremental concentrations ranging from 10^-9^ to 10^-5^ M in the absence and presence of preconstriction with U46619 (Zhou et al., 2013b). To assess the involvement of different purinergic receptors in the vasodilator response to Up_4_A, coronary small arteries from Sham and AoB swine were pre-incubated with non-selective P1 receptor antagonist 8PT (10^-5^ M), non-selective P2 receptor antagonist PPADS (10^-5^ M), adenosine A_2A_ receptor antagonist SCH58261 (10^-8^ M), P2X_1_ receptor antagonist MRS2159 (3 × 10^-5^ M), and P2Y_1_ receptor antagonist MRS2179 (10^-6^ M) followed by preconstriction with U46619 (100 nM) (Zhou et al., 2013b) and exposed to Up_4_A (10^-9^ to 10^-5^ M). To investigate if the role of endothelium-derived factors in the vasodilator response to Up_4_A was altered after AoB, vessels from both Sham and AoB were exposed to Up_4_A (10^-9^ to 10^-5^ M) in the absence and presence of nitric oxide synthase (NOS) inhibitor LNAME (10^-4^ M) alone or in combination with cyclooxygenase (COX) inhibitor indomethacin (10^-5^ M) (Zhou et al., 2013b). A potential role for TxA_2_ in the response to Up_4_A was assessed using coronary small arteries from porcine hearts (*n* = 5) obtained from a local slaughterhouse. The response to Up_4_A of coronary small arteries of slaughterhouse pigs was similar to that of Sham-operated pigs (**Figure 2**). A subset of these vessels was de-endothelialized to mimic endothelial dysfunction of cardiovascular disease condition and exposed to the TxS inhibitor ozagrel (10^-5^ M), the P2Y_12_ receptor antagonist clopidogrel (10^-6^ M), that can be converted to its active metabolites by cytochrome P450 enzymes shown to be present in the heart (Chaudhary et al., 2009; Sangkuhl et al., 2010) or a combination of ozagrel and clopidogrel prior to exposure to Up_4_A.

### Histology

Fresh sections of anterior wall of the left ventricle were fixed by 4% buffered formaldehyde and paraffin-embedded for histological analysis of remodeling of coronary small arteries. Sections were stained with resorcin-fuchsin as an elastin stain, photographed and inner and outer area of the coronary small arteries was assessed by planimetry (Sorop et al., 2016). Only transversely cut vessels with an inner radius below 200 μm were analyzed. Assuming circularity of the vessels, inner and outer radius were calculated as r = √(area/π). Wall to lumen ratio was calculated as (outer–inner diameter)/inner diameter.

### Quantitative Real-Time PCR Analysis

Following dissection, coronary small arteries (diameter: ∼150 μm) were snap-frozen in liquid nitrogen to be used for detection of purinergic receptor subtypes A_1_, A_2A_, A_3_, P2X_1_, P2X_4_, P2X_7_, P2Y_1_, P2Y_2_, P2Y_4_, P2Y_6_, and P2Y_12_ mRNA. In addition, the expression of endothelial NOS (eNOS), cyclooxygenase (COX) 1, COX2, prostacyclin synthase (PGIS), and TxS were measured (Rondelet et al., 2003). Total RNA was extracted from 5 to 7 frozen samples per group using a Qiagen RNA kit. cDNA was synthesized from 100 ng of total RNA with iScript Reverse Transcriptase (Bio-Rad). Quantitative real-time PCR (MyIQ, Bio-Rad) was performed with SYBR Green (Bio-Rad) (Zhou et al., 2013b). Target gene mRNA levels were expressed relative to the housekeeping gene glyceraldehyde-3-phosphate dehydrogenase (GAPDH) as an endogenous control (Martino et al., 2011). The primer sequences are shown in **Table 1**.

**Table 1:** Primer information.

Receptors	Sequence		Size (bp)
A_1_	5′-CCTGGCCATGCTGGCAATTGC-3′	5′-GAAGGAGAGAACCCAGCAGCC-3′	251
A_2A_	5′-ATGTTGGGCTGGAATAGCTG-3′	5′-CACGGAGTTGGTGTGAGAGA-3′	426
A_3_	5′-TACCTGCGGGTCAAGCTCACG-3′	5′-CCAGGAATGACACCAGCCAGC-3′	97
P2X_1_	5′-TTGAACCCCATTTCTTCCTG-3′	5′-AGTGCACCACACATCTGCTC-3′	248
P2X_4_	5′-TGTCCCCAGGCTACAATTTC-3′	5′-GGCAGCTTTTTCTCCCTTCT-3′	373
P2X_7_	5′-CTTTTGCACCTTGAGCTTCC-3′	5′-TCCATGCTAAGGGATTCTGG-3′	152
P2Y1	5′-TTCCTGACTTGCATCAGTGC-3′	5′-CAGTGCCCGAGTAGAAGAGG-3′	157
P2Y_2_	5′-GTGGCCTACAGCTTGGTCAT-3′	5′-GCGTGCGGAAGGAGTAGTAG-3′	235
P2Y_4_	5′-GACTGCCGGTTTAATGAGGA-3′	5′-AGGAAAAGGACGCTGCAGTA-3′	302
P2Y_6_	5′-CTGCTCTTGCCACCTGTGTA-3′	5′-AGGTTGGCGTAGAACAGGAA-3′	251
P2Y_12_	5′-AGTGATGCCAAACTGGGAAC-3′	5′-TGAATGCCCAGATAACCACA-3′	208
COX1	5′-GGAGTTTGTCAATGCCACCT-3′	5′-GCAACTGCTTCTTCCCTTTG-3′	215
COX2	5′-GGCTGCGGGAACATAATAGA-3′	5′-GCAGCTCTGGGTCAAACTTC-3′	183
PGIS	5′-CATGCGTGCTCTGATTCACT-3′	5′-AAGCTGATGCAAAGGCAAGT-3′	233
TxS	5′-AGCAAGCAGCAGAAGAGAGG-3′	5′-TCAGAGGCTTGGACAGAGGT-3′	180
eNOS	5′-CTCTCCTGTTGGCCTGACCA-3′	5′-CCGGTTACTCAGACCCAAGG-3′	151
GAPDH	5′-TCGGAGTGAACGGATTTG-3′	5′-CCTGGAAGATGGTGATGG-3′	219


### Data Analysis and Statistics

Hemodynamic data were averaged over a time period of at least 10 s. Vascular contractions were normalized to the response to 100 mM KCl, while vascular relaxation to Up_4_A was expressed as percentage of contraction to U46619 (Zhou et al., 2013b). Statistical comparison of hemodynamic data, purinergic receptor expression, vascular response to KCl and comparison of wall to lumen ratio of vessels < 200 μm between Sham and AoB swine were performed using unpaired *t*-tests. The effect of AoB on wall to lumen ratio was analyzed with ANCOVA, using inner or outer radius as covariant. The effects of AoB as well as drug treatment on the Up_4_A response were assessed using two-way ANOVA for repeated measures. Statistical significance was accepted when *P* < 0.05 (two-tailed). Data are presented as means ± SEM.

## Results

### Characteristics of AoB Animals

**FIGURE 1:**
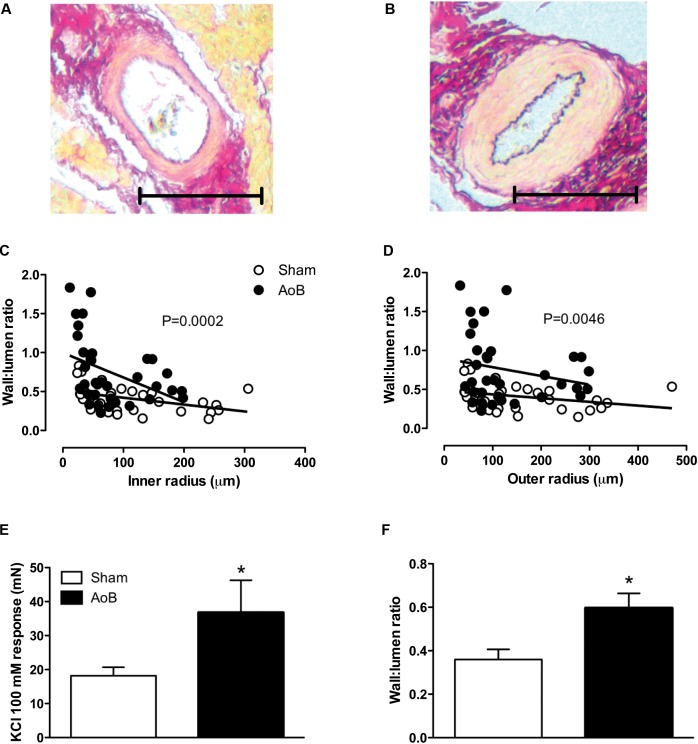
Typical examples of coronary small arteries within the myocardium of Sham-operated swine **(A)** and swine with AoB **(B)**. The scale bar denotes 100 μm. The wall to lumen ratio both as a function of inner **(C)** and outer **(D)** was significantly increased in myocardium from swine with AoB. Shown are 34 vessels from 4 Sham-operated swine and 36 vessels from 5 AoB swine. The vasoconstrictor response to 100 mM KCl was significantly increased in vessels from AoB (*n* = 6) as compared to Sham (*n* = 8), **(E)**. The wall to lumen ratio in vessels with inner diameters ranging from 100 to 200 μm was increased in AoB as compared to Sham **(F)**. ^∗^*P* < 0.05 AoB vs. Sham.

Aortic banding of the ascending aorta resulted in a systolic pressure gradient of 88 ± 2 mmHg as measured during surgery (**Table 2**). The systolic pressure gradient was stable over the time course of the experiment, as evidenced by a systolic pressure gradient of 82 ± 8 and 98 ± 6 mmHg at week 1 and week 3 after AoB (**Table 2**). Mean arterial pressure distal to the band was lower in AoB as compared to Sham during surgery and 1 week after surgery, but was similar to mean arterial pressure in Sham-operated swine 3 weeks after surgery. At end of follow-up, the HR, mean arterial pressure, and CO were comparable between Sham and AoB swine, while the PAP was slightly increased in AoB (**Table 2**). Eight weeks of AoB resulted in left ventricular hypertrophy as evidenced by a 56% increase in left ventricle weight to BW ratio (**Table 2**). The wall to lumen ratio of coronary small arteries was also increased (**Figures 1A,B** for typical examples, **Figures 1C,D** for all results, **Figure 1F** for vessels with an inner diameter ranging from 100 to 200 μm) and the vascular response to 100 mM KCl in the coronary small arteries from AoB was significantly greater as compared to that from Sham (**Figure 1E**). These observations indicate that during 8 weeks of AoB, swine develop left ventricular hypertrophy as well as medial hypertrophy of the coronary microvessels.

**Table 2:** Anatomic and hemodynamic variables.

		Sham	AoB
		*N* = 8	*N* = 7
MAP (mmHg)	Surgery	84 ± 6	56 ± 3*
	Week 1	92 ± 2	83 ± 2*
	Week 3	88 ± 2	90 ± 3
LVSP (mmHg)	Surgery	–	155 ± 5
	Week 1	–	176 ± 8
	Week 3	–	188 ± 14
SAP (mmHg)	Surgery	99 ± 6	67 ± 4*
	Week 1	112 ± 2	100 ± 2*
	Week 3	105 ± 2	104 ± 3
Pressure gradient (mmHg)	Surgery	–	88 ± 2
	Week 1	–	82 ± 8
	Week 3	–	98 ± 6
**At end of follow-up**			
BW (kg)		45 ± 0.9	42 ± 1.1
LVW/BW (g kg^-1^)		2.3 ± 0.1	3.6 ± 0.1*
HR (beats min^-1^)		109 ± 3	108 ± 4
MAP (mmHg)		98 ± 5	104 ± 3
CO (L min^-1^)		4.0 ± 0.2	4.2 ± 0.1
PAP (mmHg)		19 ± 1	23 ± 1*


*AoB, aortic banding; MAP, mean arterial pressure measured in the descending aorta; LVSP, left ventricular systolic pressure measured proximal to the band; SAP, systolic artery pressure measured in the descending aorta; BW, body weight; LVW, left ventricular weight; HR, heart rate; CO, cardiac output; PAP, pulmonary arterial pressure. Those values were obtained under anesthesia at the end of follow-up. Values are mean ± SEM; ^∗^*P* < 0.05 vs. Sham.*

### Up_4_A-Induced Coronary Relaxation Is Blunted in AoB

**FIGURE 2:**
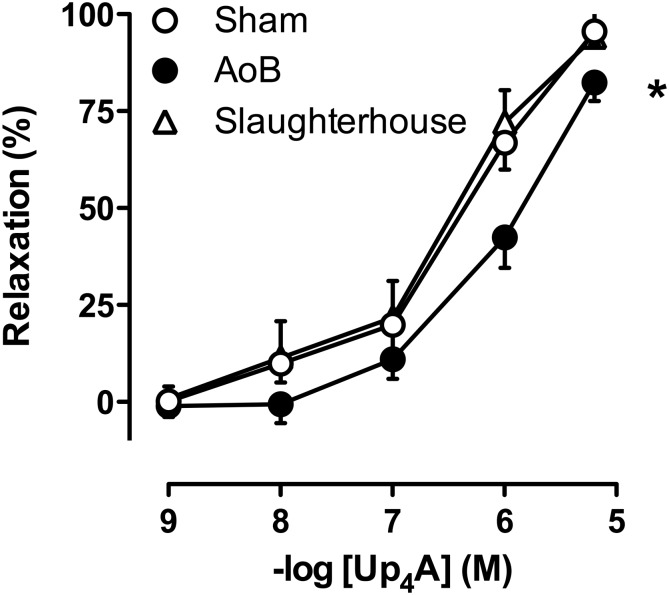
Reduced vasodilator responses to Up_4_A in coronary small arteries from swine with AoB (*n* = 6) as compared to Sham-operated (*n* = 8) or Slaughterhouse (*n* = 5) swine. ^∗^*P* < 0.05 AoB vs. Sham or Slaughterhouse.

Cumulative concentrations of Up_4_A (10^-9^ to 10^-5^ M) failed to induce vascular contraction in coronary small arteries from either Sham or AoB swine. The vasoconstrictor effect of U46619 (normalized to 100 mM KCl) was not significantly different between Sham and AoB (89 ± 15 vs. 61 ± 9%, *P* = 0.16). In these preconstricted vessels, Up_4_A produced concentration-dependent relaxation, but the relaxation was significantly less in vessels from AoB as compared to Sham (**Figure 2**).

### Involvement of Purinergic Receptor Subtypes

**FIGURE 3:**
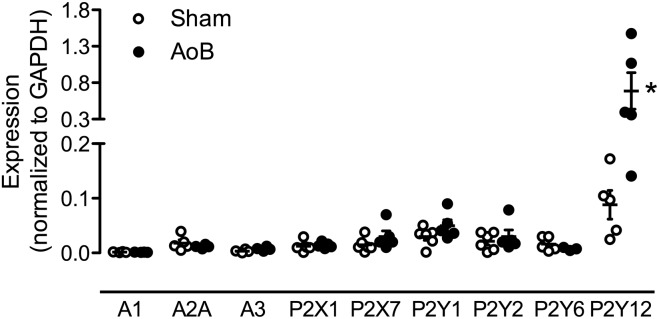
mRNA expression of various P receptor subtypes in coronary small arteries from Sham-operated swine and swine with AoB. mRNA expression was normalized to expression of GAPDH. ^∗^*P* < 0.05 vs. Sham.

**FIGURE 4:**
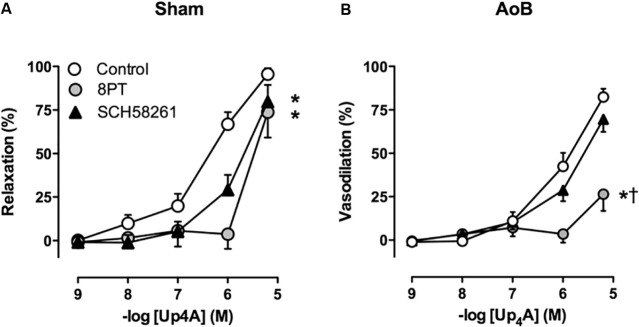
P1 receptor blockade with 8PT attenuated relaxation to Up_4_A less in coronary small arteries from Sham-operated swine (**A**, *n* = 6) as compared to coronary small arteries from swine with AoB (**B**, *n* = 6). A_2A_ receptor blockade with SCH58261 attenuated Up_4_A-induced relaxation in coronary small arteries from Sham (*n* = 5), but not AoB (*n* = 5). Control data are the same as in **Figure 2** (‘Sham’ and ‘AoB’). ^∗^*P* < 0.05 effect of 8PT vs. corresponding control. ^†^*P* < 0.05 effect of 8PT vs. corresponding SCH58261.

**FIGURE 5:**
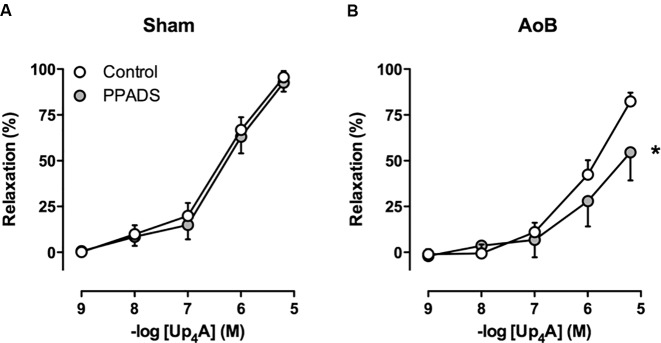
P2 receptor blockade with PPADS had no effect on relaxation to Up_4_A in coronary small arteries from Sham-operated swine (**A**, *n* = 8) but attenuated relaxation in coronary small arteries from swine with AoB (**B**, *n* = 7). Control data are the same as in **Figure 2** (‘Sham’ and ‘AoB’). ^∗^*P* < 0.05 effect of PPADS vs. corresponding control.

**FIGURE 6:**
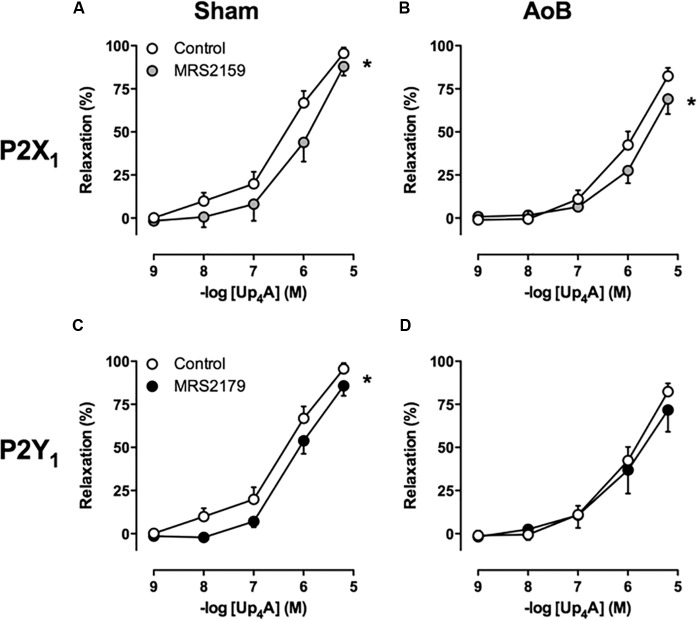
P2X_1_ receptor blockade with MRS2159 attenuated relaxation to Up_4_A to the same extent in coronary small arteries from Sham-operated swine (**A**, *n* = 8) and from swine with AoB (**B**, *n* = 6), whereas and P2Y_1_ receptor blockade with MRS2179 attenuated relaxation to Up_4_A in coronary small arteries from Sham-operated swine (**C**, *n* = 7) but not from swine with AoB (**D**, *n* = 5). Control data are the same as in **Figure 2** (‘Sham’ and ‘AoB’).^∗^*P* < 0.05 effect of MRS2159 or MRS2179 vs. corresponding control.

Despite unaltered expression of the A_1_, A_2A_, and A_3_ receptor (**Figure 3**), P1 receptor blockade with 8PT attenuated Up_4_A-induced relaxation in coronary small arteries from AoB more than in Sham (**Figures 4A,B**). Conversely, A_2A_ receptor blockade with SCH58261 attenuated Up_4_A-induced relaxation to the same extent as 8PT in coronary small arteries from Sham, but had no effect on Up_4_A-induced relaxation in AoB (**Figures 4A,B**). Non-selective P2 receptor blockade with PPADS had no effect on Up_4_A-induced relaxation in coronary small arteries from Sham (**Figure 5A**), but did attenuate Up_4_A-induced relaxation in vessels from AoB (**Figure 5B**). P2X_1_ receptor blockade with MRS2159 attenuated Up_4_A-induced relaxation in coronary small arteries from Sham (**Figure 6A**) and AoB swine (**Figure 6B**) to a similar extent, whereas P2Y_1_ receptor blockade with MRS2179 attenuated Up_4_A-induced relaxation in coronary small arteries from Sham (**Figure 6C**), but not AoB swine (**Figure 6D**). P2X_1_, P2X_7_, P2Y_1_, P2Y_2_ and P2Y_6_ receptors were expressed, but no differences in expression in coronary small arteries between Sham and AoB were found (**Figure 3**). Expression of P2Y_12_ receptors was higher in coronary small arteries from AoB (**Figure 3**), while expression of P2X_4_ and P2Y_4_ could not be detected (data not shown).

### Involvement of Endothelium-Derived Factors in the Response to Up_4_A

**FIGURE 7:**
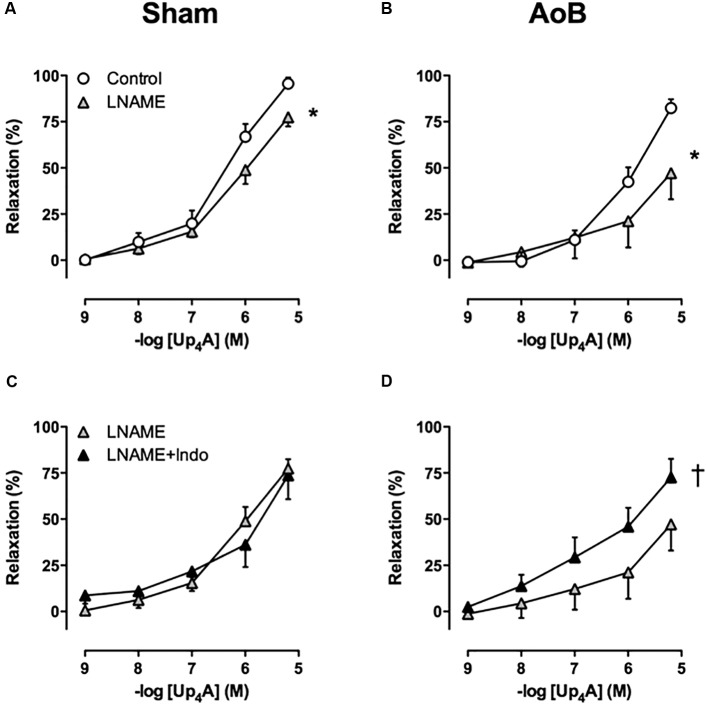
eNOS inhibition with LNAME attenuated the response to Up_4_A to a similar extent in coronary small arteries from Sham-operated swine (**A**, *n* = 8) and swine with AoB (**B**, *n* = 7). Subsequent inhibition of cyclooxygenase with indomethacin (Indo) had no effect in vessels from Sham (**C**, *n* = 7), but enhanced relaxation to Up_4_A in vessels from swine with AoB (**D**, *n* = 6). Control data are the same as in **Figure 2** (‘Sham’ and ‘AoB’). ^∗^*P* < 0.05 effect of LNAME vs. corresponding control; ^†^*P* < 0.05 effect of LNAME + indomethacin vs. LNAME alone.

**FIGURE 8:**
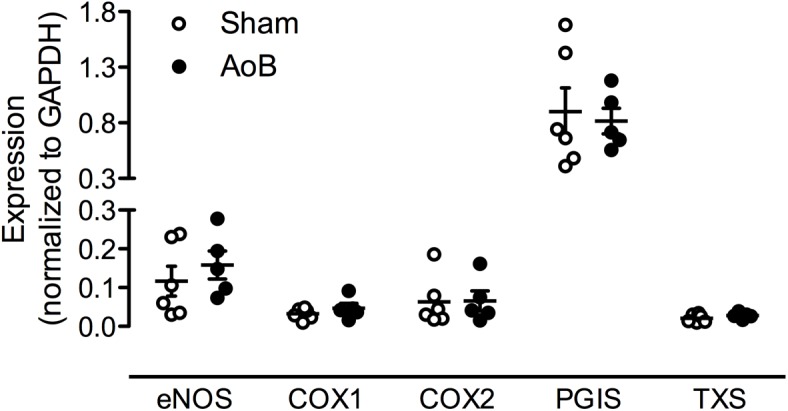
mRNA expression of eNOS, cyclooxygenase (COX) 1 and COX2, prostacycline synthase (PGIS) and TxS in coronary small arteries from Sham-operated swine and swine with AoB. mRNA expression was normalized to expression of GAPDH.

To investigate the contribution of alterations in endothelial function to the attenuated response to Up_4_A after AoB, the effects of eNOS and COX inhibition on Up_4_A-induced relaxation were assessed. eNOS inhibition with LNAME attenuated Up_4_A-induced relaxation to the same extent in coronary small arteries from Sham and AoB swine (**Figures 7A,B**), which was corroborated by similar eNOS expression level in vessels from AoB and Sham (**Figure 8**).

**FIGURE 9:**
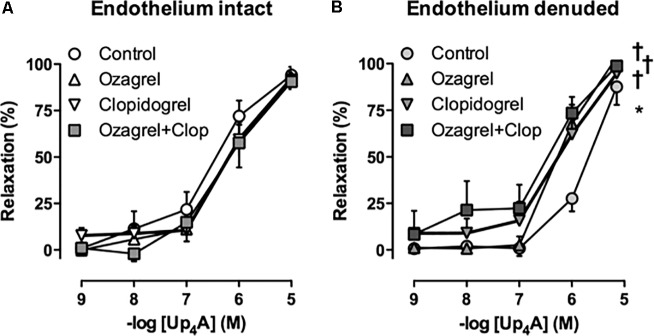
Neither TxS inhibition with ozagrel, nor P2Y_12_ receptor blockade with clopidogrel (Clop), nor the combination affected the response to Up_4_A in coronary small arteries from slaughterhouse swine with intact endothelium (**A**, *n* = 5). Ozagrel and clopidogrel enhanced vasodilator response to Up_4_A in denuded coronary small arteries, while the effect of the combination of ozagrel and clopidogrel was similar to the effect of either inhibitor alone (**B**, *n* = 5). Control data are the same as in **Figure 2** ‘Slaughterhouse.’ ^∗^*P* < 0.05 effect of denudation; †*P* < 0.05 effect of ozagrel and/or clopidogrel vs. corresponding control.

In Sham-operated swine, combined inhibition of eNOS and COX (LNAME+indomethacin) attenuated Up_4_A-induced relaxation to the same extent as LNAME alone (**Figure 7C**). In contrast, combined inhibition of eNOS and COX in vessels from AoB swine significantly enhanced Up_4_A-induced relaxation compared to LNAME alone (**Figure 7D**), although COX1, COX2, and PGIS expression were not different in coronary small arteries between Sham and AoB (**Figure 8**). The observation that COX-inhibition enhanced the vasodilator effect of Up_4_A suggests the production of a vasoconstrictor prostanoid(s). There is some evidence linking TxA_2_ production to P2Y_12_ receptor activation (Bhavaraju et al., 2010). Given the increased P2Y_12_ receptor expression in coronary small arteries of AoB (**Figure 3**), we further investigated if TxA_2_ could be the vasoconstrictor prostanoid produced in response to Up_4_A, as well as the functional involvement of P2Y_12_ receptor in this process. Coronary small arteries were denuded to mimic endothelial dysfunction in hypertension. Subsequently, endothelium-intact and -denuded vessels were exposed to Up_4_A in the presence of the TxS inhibitor ozagrel, the P2Y_12_ receptor antagonist clopidogrel or their combination. Ozagrel had no effect on the response to Up_4_A in coronary small arteries with intact endothelium (**Figure 9A**), but enhanced the vasodilator response to Up_4_A in denuded coronary small arteries (**Figure 9B**). Similarly, clopidogrel enhanced the vasodilator response to Up_4_A in denuded coronary small arteries (**Figure 9B**), but not in coronary small arteries with intact endothelium (**Figure 9A**). As the effect of the combination of ozagrel and clopidogrel was identical to the effect of either ozagrel or clopidogrel alone (**Figure 9B**), these data are consistent with the concept that P2Y_12_ receptor activation may induce TxA_2_ production.

## Discussion

The main findings of the present study were that (i) AoB resulted in medial hypertrophy of coronary small arteries as evidenced by an increase in wall-to-lumen ratio and an increased KCl-induced contractile force. (ii) Up_4_A-induced relaxation was reduced in coronary small arteries from AoB as compared to Sham. (iii) Blockade of P1 receptors attenuated Up_4_A-induced relaxation less in coronary small arteries from Sham as compared to AoB, but the contribution of the A_2A_ receptor was reduced. (iv) P2 receptor blockade with PPADS attenuated Up_4_A-induced relaxation in AoB but not Sham. (v) The response to P2X_1_ blockade was similar in coronary small arteries from AoB and Sham. (vi) P2Y_1_ receptor blockade attenuated Up_4_A-induced relaxation in Sham, but not AoB. (vii) Expression of the P2Y_12_ receptor was increased in coronary small arteries from AoB, while expression of other purinergic receptor subtypes involved in vascular tone regulation was not altered. (viii) eNOS inhibition attenuated Up_4_A-induced relaxation to the same extent in Sham and AoB, whereas additional COX inhibition had no effect in Sham, but enhanced Up_4_A-induced relaxation in AoB. (ix) P2Y_12_ receptor blockade and/or TxS inhibition enhanced the vasodilator response to Up_4_A in denuded coronary small arteries. The implications of these findings are discussed below.

Consistent with previous reports (Desjardins et al., 2005; Aubin et al., 2007), 8 weeks of AoB resulted in left ventricular hypertrophy and coronary microvascular remodeling, as evidenced by doubling of wall to lumen ratio of the coronary small arteries and doubling of the contractile response to KCl. The vasodilator response to Up_4_A was blunted in coronary small arteries from swine with AoB. Up_4_A exerts its vasoactive effect through interaction with purinergic receptors. In accordance with previous studies from our laboratory (Zhou et al., 2013b), coronary relaxation induced by Up_4_A in swine was mediated primarily by the P1 receptors. Although the expression of the A_1_, A_2A_, and A_3_ receptors was unaltered after AoB, the overall contribution of the P1 receptors to Up_4_A-induced vasorelaxation was increased, while contribution of the A_2A_ receptor was reduced. These findings are different from our findings in the porcine coronary vasculature after MI (Zhou et al., 2013a) and metabolic derangement (Zhou et al., 2017), in which the reduced response to Up_4_A was not due to functional loss of A_2A_ receptors. Although studies in mice (Zhou et al., 2015a) and swine (Long et al., 2010) showed that the main vasoactive effect of adenosine was mediated through A_2A_, rather than A_2*B*_ receptor, we cannot exclude that altered expression of the A_2*B*_ receptor could explain the increased contribution of P1 receptors to the vasorelaxation to Up_4_A in coronary small arteries from AoB as compared to Sham.

The assessment of the contribution of the P2 receptors to the response to Up_4_A is difficult due to lack of selective antagonists of many of the P2 receptor subtypes. In general, activation of P2 receptors on endothelial cells is thought to result in vasodilation, whereas activation of P2 receptors on vascular smooth muscle cells results in vasoconstriction (Matsumoto et al., 1997; Burnstock, 2010; Zhou et al., 2013b). The non-selective P2 antagonist PPADS attenuated Up_4_A-induced relaxation in coronary small arteries from swine with AoB, but not from Sham-operated swine, indicating that the contribution of P2 receptors blocked by PPADS is altered. PPADS has been shown to block P2X_2_, P2X_3_, P2X_5_ (Burnstock, 2007), P2X_7_ (Mezzaroma et al., 2011), P2Y_1_ (Ju et al., 2003), P2Y_2_, P2Y_4_ (Rayment et al., 2007), and P2Y_6_ (Schreiber and Kunzelmann, 2005) receptors. In contrast to the altered effect of PPADS, blockade of P2X_1_ receptors attenuated the vasodilator response to Up_4_A to the same extent in coronary small arteries from AoB and Sham-operated swine, which is in accordance with its unaltered expression. Conversely, despite unaltered expression of the P2Y_1_ receptor, its vasodilator effect in response to Up_4_A that was present in coronary small arteries from Sham disappeared after AoB. The observation that, in vessels from Sham-operated swine, P2Y_1_ blockade attenuated the response to Up_4_A, whereas PPADS, that has also been shown to block the P2Y_1_ receptor, does not, suggests that PPADS also blocks a P2 vasoconstrictor receptor. The exact identity of this receptor remains to be elucidated. Interestingly, expression of the P2Y_12_ receptor was increased in coronary small arteries from swine with AoB. As activation of the P2Y_12_ receptor on vascular smooth muscle cells results in vasoconstriction (Wihlborg et al., 2004), increased expression of this receptor could explain the reduced vasodilator effect of Up_4_A in coronary small arteries from swine with AoB. A role for the P2Y_12_ receptor in the reduced vasodilator response to Up_4_A is further substantiated by our observation that P2Y_12_ blockade with clodipogrel enhanced the vasodilator response to Up_4_A in denuded vessels, although it had no effect on the vasodilator response in vessels with intact endothelium. Altogether, our data indicate that Up_4_A-mediated activation of the P2Y_12_ receptor on the vascular smooth muscle cells results in vascular contraction, while the presence of healthy endothelium prevents such response.

Since several studies have shown endothelial dysfunction in the porcine coronary vasculature following AoB (Malo et al., 2003a,b; Desjardins et al., 2005; Aubin et al., 2007), we further investigated whether the contribution of endothelial vasodilator pathways to Up_4_A-induced relaxation was altered. The blunted response to Up_4_A was not due to a decreased contribution of NO, as both the effect of eNOS-inhibition with LNAME and eNOS expression were similar in coronary small arteries from Sham-operated swine and swine with AoB. The unaltered expression of eNOS is consistent with another study in isolated coronary arteries from swine with AoB (Malo et al., 2003b). Also, the contribution of eNOS to bradykinin-induced relaxation was maintained (Aubin et al., 2007), despite the presence of eNOS uncoupling (Malo et al., 2003b).

Cyclooxygenase-inhibition with indomethacin potentiated the vasodilator response to Up_4_A in vessels from AoB, but not Sham-operated animals, suggesting that the reduced responsiveness to Up_4_A was, at least in part, due to production of a vasoconstrictor prostanoid(s). A shift in the balance from vasodilator prostanoids to vasoconstrictor prostanoids has been implicated in the pathogenesis of cardiovascular disease (Kawabe et al., 2010). A potential mechanism behind such shift may be that oxidative stress and/or endothelial dysfunction result in eNOS uncoupling, which subsequently leads to the production of peroxynitrite, that is capable of inactivating PGIS thereby causing a shift in production from prostacyclin to TxA_2_ (Zou et al., 2004; Nie et al., 2006). Indeed, TxA_2_ levels were increased in the myocardium of hypercholesterolemic swine with endothelial dysfunction (Chu et al., 2012). Furthermore, the presence of a healthy endothelium in the present study prevented production of TxA_2_, as inhibition of TxS with ozagrel enhanced the vasodilator response to Up_4_A in denuded coronary small arteries, but not in those with intact endothelium. A link between the vasoconstrictor effect of Up_4_A and TxA_2_ production is further supported by a recent study demonstrating that Up_4_A stimulates TxA_2_ production leading to vascular contraction in mouse aortas (Zhou et al., 2015b). Moreover, an enhanced contraction in response to Up_4_A is mediated through activation of COX2 and production of TxA_2_ in the renal vasculature of diabetic rats (Matsumoto et al., 2014). Although mRNA levels of COX1, COX2, PGIS, and TxS were unaltered in coronary small arteries from swine with AoB in the present study, it is possible that the *activity* of COX2 and/or TxS was increased following stimulation with Up_4_A. Preliminary data from coronary small arteries from two swine with AoB show that inhibition of TxS with ozagrel after LNAME has a similar effect as indomethacin, suggesting that indeed, TxS activity is increased. An increased production of TxA_2_ in the coronary vasculature in AoB is not unique to Up_4_A, but has also been shown in response to bradykinin and serotonin (Desjardins et al., 2005). It is unlikely that the response of the coronary microvasculature to TxA_2_ receptor activation was altered, as the response to the TxA_2_ analog U46619 was identical in vessels from Sham and AoB swine.

There is some evidence linking TxA_2_ production to activation of the P2Y_12_ receptor. Thus, TxA_2_ levels were reduced in human serum treated with P2Y_12_ inhibitors, P2Y_12_ inhibition reduced serum TxA_2_ in mice, and serum TxA_2_ levels were reduced in P2Y_12_ knockout mice (Bhavaraju et al., 2010). Consistent with these observations, in the present study, P2Y_12_ blockade enhanced the vasodilator response to Up_4_A to a similar extent as TxS inhibition, and combined inhibition of P2Y_12_ receptor and TxS had no additional effect as compared to each treatment alone.

A role for Up_4_A in the pathogenesis of hypertension has been suggested by the observation that Up_4_A-induced vascular contraction in the renal, femoral, and basilar artery is potentiated in hypertension (Matsumoto et al., 2011b), while the Up_4_A plasma concentration in hypertensive subjects is significantly higher as compared to that in healthy subjects and correlates with blood pressure (Jankowski et al., 2007). The present study shows that pressure overload-induced coronary vascular remodeling results in attenuation of the vasodilator effect of Up_4_A, which is accompanied by increased expression of P2Y_12_ receptor. Activation of the P2Y_12_ receptor on vascular smooth muscle likely results in activation of TxS and TxA_2_ production in response to Up_4_A, thereby blunting its vasodilator effect in the coronary microcirculation. Future experiments are required to investigate if indeed the P2Y_12_ receptor is a key factor in activation of TxS in AoB animals, as well as the signal transduction pathway involved.

## Author Contributions

ZZ: designed and performed the experiments, interpreted the data, drafted the manuscript. IL: performed the experiments, drafted the manuscript. HvB and CC: interpreted the data, revised the manuscript. DD and DM: designed the experiments, interpreted the data, revised the manuscript.

## Conflict of Interest Statement

The authors declare that the research was conducted in the absence of any commercial or financial relationships that could be construed as a potential conflict of interest.

## Abbreviations

AoB: aortic banding
BW: body weight
CO: cardiac output
HR: heart rate
LNAME: N^ω^-nitro-L-arginine methyl ester HCl
LVSP: left ventricular systolic pressure measured proximal to the band
LVW: left ventricular weight at end of follow-up
MAP: mean arterial pressure measured in the descending aorta
NO: nitric oxide
PAP: pulmonary arterial pressure
PGI2: prostacyclin
PGI2S: prostacyclin synthase
PPADS: pyridoxalphosphate-6-azophenyl-2′,4′-disulfonic acid
P1-receptors: purinergic type 1 receptors
P2-receptors: purinergic type 2 receptors
SAP: systolic artery pressure measured in the descending aorta
TxS: thromboxane synthase
Up_4_A: uridine adenosine tetraphosphate
U46619: 9,11-dideoxy-9α,11α-methanoepoxy Prostaglandin F2α
8PT: 8-phenyltheophylline
